# A phenotype-based ageing clock that reflects development rather than senescence in a wild mammal

**DOI:** 10.1098/rstb.2025.0327

**Published:** 2026-11-12

**Authors:** Lucy Barnard, Erin R. Siracusa, Xavier Bal, Jill G. Pilkington, Josephine Pemberton, Jon Slate, Daniel H. Nussey

**Affiliations:** ^1^ School of Biosciences, The University of Sheffield, Sheffield, UK; ^2^ Centre for Research in Animal Behaviour, University of Exeter, Exeter, UK; ^3^ Institute of Ecology and Evolution, School of Biological Sciences, The University of Edinburgh, Edinburgh, UK

**Keywords:** ageing clock, development, ageing, development clock, senescence, Soay sheep (*Ovis aries*)

## Abstract

Epigenetic ageing clocks accurately predict chronological age (CAge) across species. The deviation between their age predictions and actual CAge (Δage) is an established metric of senescence and predictor of mortality in humans. In field-based studies of wild animals, epigenetic clocks can be challenging to construct. A possible alternative is to build clocks from phenotypic data that are routinely collected in such studies. Here, we tested a phenotypic ageing clock based on extensive morphological, behavioural, and physiological data from a wild Soay sheep population. Clocks constructed from morphological data predicted age with the greatest accuracy. Their predictive power was comparable to published epigenetic clocks until advanced adulthood, after which it declined. Δage varied with early environmental conditions and birth weight. Heavier-born animals tended to have greater Δage, implying the clock may reflect developmental rate. Greater Δage in adulthood was associated with increased subsequent over-winter survival. Our results suggest that phenotypic ageing clocks can carry a strong developmental signal. Accordingly, they may reflect early life conditions and subsequent fitness in an opposite way to epigenetic ageing clocks, which capture senescence. Further studies are required to test whether our findings generalize to other systems and types of phenotypic measurements.

This article is part of the theme issue ‘Developmental circuits in ageing’.

## 1. Introduction

Ageing describes changes in physiological function, phenotype, and demography that occur across the lifetime of an individual. It is most commonly used in relation to the deterioration in physiological function observed in later adulthood, or senescence. Growing evidence from various disciplines, including bio-gerontology, evolution, and ecology, suggests close links between developmental processes and subsequent senescence. Early life experiences and developmental genes and pathways appear to shape variation in adult function and fitness [1,2]. To understand these links between early and later life, we need to measure individual trajectories in biological state or function across the entire life course. Chronological age (CAge) alone offers limited insight, as it is biologically invariant and individuals of the same age are often in very different stages of development or senescence.

In bio-gerontology, an individual's ‘biological age’ refers to their functional state, independent of their CAge. Biological ageing clocks (BACs) are the current gold standard to measure this state in adult humans [3]. BACs apply machine learning to large-scale ‘omics’ or medical data to predict CAge or health outcomes. Human clocks built using epigenetic or frailty data predict CAge with great accuracy [4–6]. Deviations of BAC predictions from CAge (∆age) are widely used as proxies for biological age and predict important health outcomes and follow-up mortality risk [4,5,7] (see figure 1). Individuals experiencing adverse early life conditions also tend to have greater BAC age predictions (positive ∆age; figure 1), suggesting faster senescence in those experiencing poor developmental conditions [8]. Wider application of BACs is essential for enhancing our understanding of ageing processes and their developmental drivers across diverse ecological contexts.

**Figure 1.**
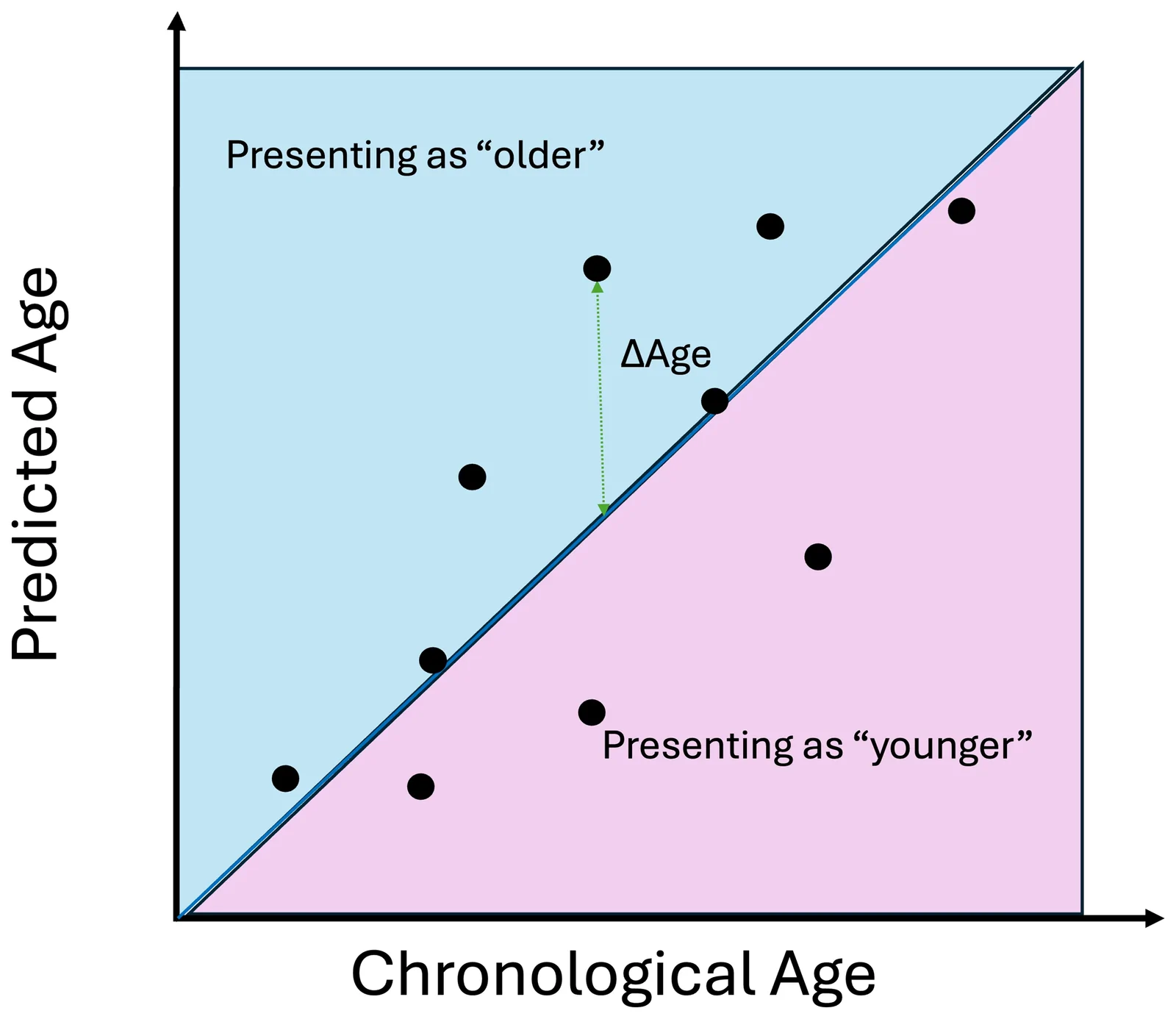
Illustration of the application of biological ageing clocks to estimate ∆age. The clock applies machine learning algorithms, such as elastic net regression, to large-scale data to predict age as accurately as possible. The plot shows this prediction, with the black line indicating the 1 : 1 relationship of chronological and predicted age. Individual predictions of age from the clock deviate from their actual chronological age. Those predictions below the fit line can be viewed as biologically or phenotypically ‘younger’ (pink area) and those above as biologically or phenotypically ‘older’ (blue area). The deviation can be measured for each sample (∆age) and used as an indicator of biological age.

Individual-based field studies of wild vertebrates are ideal systems to test how development shapes adult senescence, health, and fitness. Such studies monitor and sample known individuals across much of their natural lifetimes, providing the detailed longitudinal data necessary to connect early and late life processes [9]. Importantly, wild populations experience complex and variable environmental challenges across their lifetimes. They can offer important insight into the evolutionary and environmental forces shaping senescence in uncontrolled, natural conditions [10]. Declines in demographic rates (survival and fertility) with age through later adulthood are widely observed in wild vertebrates [11]. Long-term studies further show that genes, early life adversity, and early life reproductive investment all shape the rate of demographic decline with age under natural conditions [1,12,13]. BACs offer a potentially powerful way to better predict variation in demographic rates and understand the biological processes underpinning variation in ageing in wild animals.

Epigenetic ageing clocks, based on methylation data, are the most widely applied BAC in wild mammals and birds [14,15]. Despite their evident power, there are challenges applying epigenetic clocks in the wild. They require collection of high-quality DNA samples, sequencing is costly and analysis is computationally demanding. Long-term studies often collect data on different phenotypes, including morphology, life history, and behaviour, as well as samples (most commonly blood and faeces) used to assay low-cost physiological markers (immune, endocrine measures) and parasite burdens. Many of these traits are known to vary with age. A recent study of wild baboons used such data to build a BAC which showed moderate ability to predict adult age [16]. ∆age scores from this clock behaved much like those from human epigenetic clocks. Experience of early life adversity was associated with greater ∆age, consistent with faster biological ageing. ∆age was also positively associated with increased mortality risk. This first wild animal phenotype ageing clock focused on mature adults. To date to our knowledge, no study has explored how such phenotypic BACs built using data from across the entire life course behave, or the degree to which they reflect development versus senescence or both.

Here, we used four decades of morphological, physiological, and behavioural data collected from a long-term individual-based study of wild Soay sheep (*Ovis aries*) to build a phenotypic BAC encompassing all ages. We tested the ability of these different types of data to predict age. We then tested how ∆age scores from this clock related to measures of early life environment and gestational growth, and subsequent over-winter mortality.

## 2. Methods

### (a) Study system

Soay sheep have lived unmanaged on Hirta, part of the St Kilda archipelago off the northwest coast of Scotland (57°49' N, 8°34' W), since the 1930s and on the adjacent island of Soay for several millennia. The individuals in the Village Bay area of Hirta have been the focus of long-term study since 1985 [17]. Soay sheep are a small, primitive breed of sheep with marked sexual dimorphism. Soays are gregarious with a dynamic fission–fusion society; individuals feed, shelter, and move in readily identifiable groups, showing consistent association patterns across seasons and years. They have a promiscuous mating system where males engage in intense competition for access to oestrous females. Soay sheep rut in the autumn and give birth in the spring (March–April). Females typically give birth to a single lamb, although approximately 13% of litters are twins. Although the population is free from predation risk, it experiences many environmental challenges, including food limitation, thermoregulatory challenges associated with winter weather conditions and parasite infection [17]. The majority (greater than 80%) of mortality in the Soay sheep occurs over winter (December–April), as a result of these combined pressures. First winter mortality is high among lambs (ranging between 10% and 40% depending on environmental conditions and density [18]), but females who survive to maturity can live to be up to 16 years old and males up to 12 years old.

Core data collection involves capturing, weighing, and ear tagging lambs in the Village Bay study area within a few days of birth [19]. Approximately 95% of individuals are caught and tagged, and subsequently followed until death. Each August, approximately 60% of the individuals in the study area are re-captured and processed for morphological measurements, and faecal and blood sampling which are used for parasite and immunological assays, respectively. Additionally, the population is censused 30 times per year (10 censuses each in spring, summer, and autumn). During censusing, three observers walk different fixed routes simultaneously, recording the activity and location of all individuals in the study area as well as group size and membership. Observers assign group membership using a chain-rule where all individuals within approximately 20 m of each other are deemed to be associating [20]. Age at death can be determined for most individuals, either directly through mortality searches or inferred for animals that disappear from censuses. The number of resident sheep (Village Bay population size) is estimated each October using a combination of census, birth, and mortality data. Since birth year is known for all individuals in this study and births occur in the same season each year, we have measured individual CAge in years.

### (b) Phenotypic data

We identified 32 different phenotypes from the Soay Sheep Project's long-term database that are known to, or have the potential to, change with age (supplementary material, figure S1 and table S2). Detailed descriptions of the traits, and how they were measured can be found in the supplementary material, table S1. All continuous data were mean centred and scaled by one standard deviation to create a *z*-score. Categorical variables were coded as 0/1. We separated the phenotypes into three groups based on the type of data collected and the sample sizes available. ‘Morphological’ data were measured from individuals caught in August (1985–2024 inclusive) and encompassed traits such as weight, dental information, and leg length (supplementary material, table S1). ‘Bioassay’ data were collected from faecal or blood samples taken at August captures in 1998–2015. These included parasite faecal egg counts [21], anti-parasite antibody measures of three isotypes, immunoglobuin A, E and G (IgA, IgE, and IgG) [22], and relative telomere length [23] (supplementary material, table S1). ‘Behaviour’ data included social and spatial traits derived from census data collected 1986–2023 and included measures of social integration, social bonding, and home range size (see the supplementary material, methods and table S1).

### (c) Phenotypic clock construction

We built separate clocks based on each of our three data categories and a final clock that combined all data (‘composite clock’). Because some morphological traits are only expressed in one sex, we built separate male and female clocks. Not all phenotype measurements were collected in all years, meaning each clock had different sample sizes (table 1). The best performing clock (see below for explanation of performance assessment) was then re-run on just the individuals aged 2 years and over (‘adult clock’). This was to check how well the clock could predict age after maturity (comparable to [5,16]). Soay sheep have attained adult size and are all sexually mature by 2 years of age.

**Table 1.** Phenotype clock model results. (All models had alpha set to 0.5. Lambda, Pearson correlation coefficient (*r*) between the chronological age and predicted age in each leave-one-animal-out cross-validation (LOAOCV) model, and associated *p*-value are reported. Mean squared error (MSE) and median absolute error (MAE) for each clock are reported in years. All clocks spanned the full age range of males and females (0–15 and 0–11 years, respectively), except the male bioassay (0–10 years) and the composite clocks (females: 1–14 years, males: 1–10 years). The adult morphology clocks only included sheep aged 2 years and over.)

clock	number of records	number of individuals	lambda	Pearson's *r*	*p*-value	MSE	MAE	*r*^2^	available variables	final model variable count
female morphology	3679	1603	0.009	0.914	<0.001	1.608	0.431	0.835	8	8
female bioassay	2010	823	0.012	0.554	<0.001	6.554	1.659	0.307	6	6
female behaviour	6182	1319	0.000	0.197	<0.001	8.071	2.175	0.039	15	15
female composite	1054	436	0.036	0.831	<0.001	2.339	0.733	0.689	29	19
female adult morphology	2112	730	0.009	0.780	<0.001	2.663	0.917	0.608	8	8
male morphology	1505	1078	0.005	0.948	<0.001	0.342	0.184	0.899	12	11
male bioassay	764	487	0.014	0.584	<0.001	2.673	0.755	0.341	6	6
male behaviour	1898	746	0.001	0.604	<0.001	2.456	0.929	0.365	15	14
male composite	209	124	0.042	0.870	<0.001	0.901	0.455	0.757	33	20
male adult morphology	422	228	0.039	0.817	<0.001	1.034	0.525	0.667	12	6

We used an elastic net regression (ENR) to build each clock model, using the ‘glmnet’ and ‘cv.glmnet’ functions in the glmnet package (version 4.1-10; [24]) in R (version 4.4.1). ENR is designed to be used when there are many highly correlated variables with the goal of finding the variables that carry the most information without over-parameterizing the model. The relevant phenotypic traits were fitted as the predictor variables and CAge in years was the response variable. ENR is a machine learning method that balances ridge and lasso regularization to apply shrinkage to model term coefficients, or remove them completely, to improve prediction accuracy [25]. Reducing the complexity of the model avoids overfitting to the training data. The balance between ridge regression and lasso is defined by the *⍺*-value which in this case was set to 0.5, meaning an equal contribution of each. The ENR model outputs variables with non-zero coefficients and the lambda value which indicates the strength of shrinkage that has been applied to the model coefficients.

To assess the predictive accuracy of our clocks, we used leave-one-animal-out cross-validation (LOAOCV). This allowed us to generate confidence estimates for the final clock models. LOAOCV is an extension of *k*-fold cross-validation where all records from each individual, in turn, act as the model test set while the remaining data act as the training set. Grouping records by individual reduces information leakage between the training and test sets. Since relatively few records are left out, the model produced by each iteration (one animal excluded) is expected to be very similar to the model created by the full dataset (no animals excluded). Each individual therefore has a unique model for predicting age and each measurement will have an associated predicted age (PAge). We used LOAOCV to estimate model performance through two metrics: mean squared error (MSE) and median absolute error (MAE). MAE is the median value of the difference between an individual's predicted and CAges, and indicates how accurately the model is able to predict age. MSE is the mean of all the squared errors between predicted and CAges. The overall performance of a clock was assessed through correlating the PAge and known CAge for each record.

### (d) Environmental data

To test the effects of early life conditions on phenotypic clock scores, we used a range of available measurements from the first year of individuals’ lives, detailed below. A recent study documented lasting effects of these early life measures on adult lifetime and annual fitness in the Soay sheep [26].

Birth weight: measured in kilograms when lambs were captured within a few days of birth. Since not all individuals were caught on the same day after birth, we used the residual of the regression of all available birth weight measures against age at capture (in days) for individuals less than 7 days old, following previous methodology [26]. This was done for each sex separately (females: *n* = 3050, males *n* = 2986). Lighter birth weights are associated with poor gestational conditions and predict increased over-winter mortality in the first year of life and reduced adult longevity [26].

Litter size: individuals were classed as singletons or twins. There was one instance of triplets in the dataset which was grouped with the twins. Twinning is associated with reduced growth rates and increased over-winter mortality in the first year of life [26].

Population size in year of birth: we used the estimate of Village Bay population size in October of the individual's year of birth. Greater population sizes are associated with increased over-winter mortality owing to increased resource competition, particularly for lambs [27].

Population size during gestation: as above, but for the year before birth, reflecting resource competition the mother experienced while gestating. Greater population sizes during gestation are associated with increased over-winter mortality in the first year of life [18].

Parasite exposure in year of birth: the mean strongyle faecal egg count from all August samples from lambs born that year, see [26] for full details. Lambs in cohorts with higher mean faecal egg counts are less likely to survive their first winter [26].

First winter NAO: the Hurrell North Atlantic Oscillation Index (NAO) is a measure of atmospheric pressure across the North Atlantic [26,28]. It is based on the difference in barometric pressure between Portugal and Iceland. Winter NAO (December, January, and February) predicts sheep mortality rate on St Kilda. High NAO values, reflecting wet, windy and stormy winters, are associated with higher over-winter mortality compared to low NAO values, reflecting dry, cold winters. Estimates of NAO were taken from the NCAR Climate Data Guide [29].

### (e) Calculating ∆age and testing relationships with early life conditions and survival

We calculated ∆age as the difference between an individual's phenotypic clock age and their CAge. We focussed on the morphology clock as this performed best and had the largest available sample sizes (figure 2; table 1). ∆age was calculated as:

$$\begin{array}{r} \Delta age=PAge-CAge. \end{array}$$

**Figure 2.**
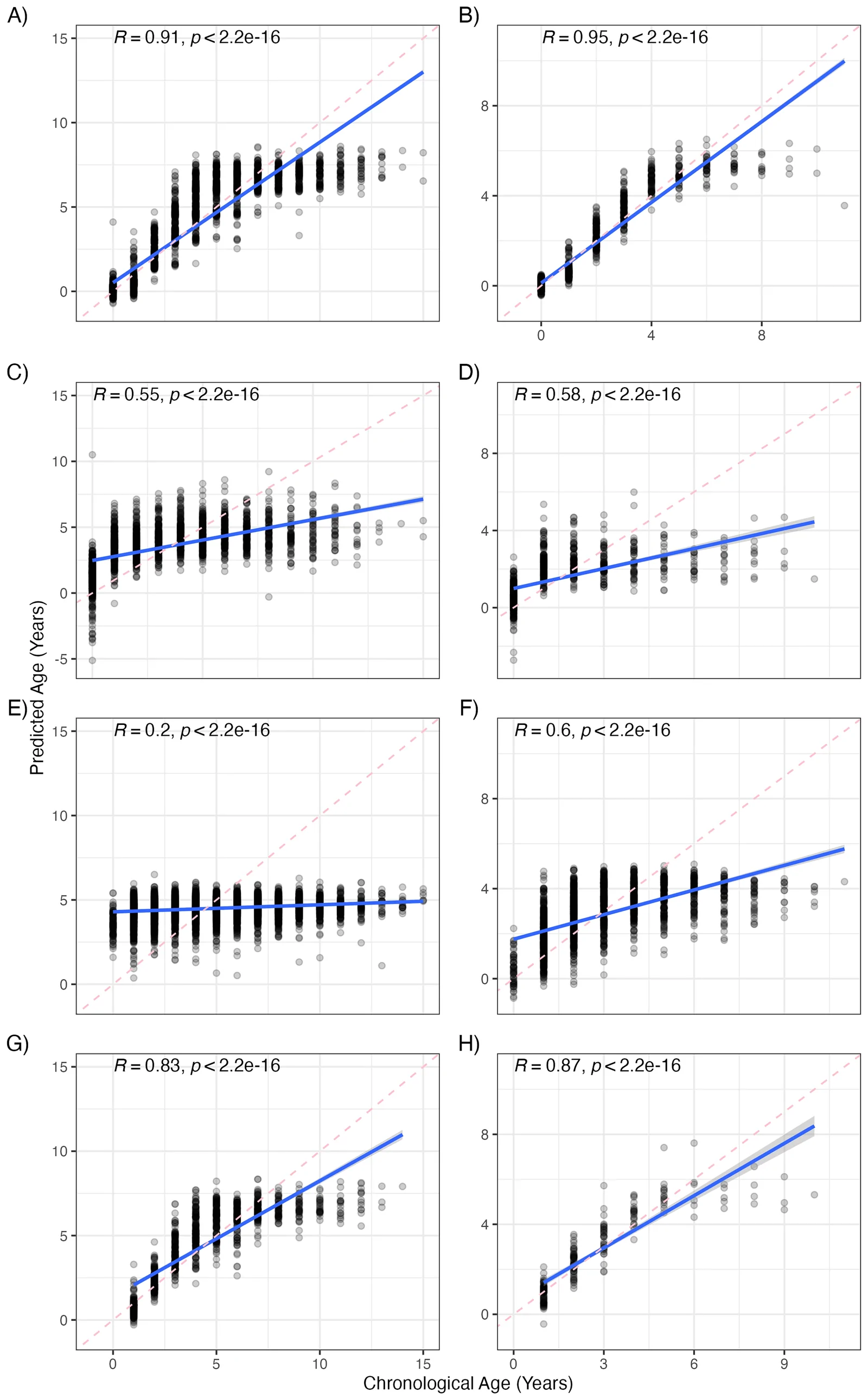
The relationship between phenotypic and chronological age. (A) Morphology clock, female, (B) morphology clock, male, (C) bioassay clock, female, (D) bioassay clock, male, (E) behaviour clock, female, (F) behaviour clock, male, (G) composite clock, female, (H) composite clock, male. Solid line is line of best fit, dashed line is *y* = *x*, shading is the standard error.

We calculated the repeatability of ∆age in these models using the ‘RptR’ package [30]. To investigate the effect of early life conditions on adult ∆age, we fitted linear mixed effects models with a Gaussian distribution, using only individuals aged 2 years or more, with separate models for each sex. Modelling was done on all ∆age measures from individuals which had an available birth weight and first winter NAO (females: *n* = 1548, males: *n* = 293). In all models, we included ∆age as our response variable, and all the early life conditions measures described above as fixed effects. Fixed effects were standardized to a mean of zero and standard deviation of one for comparison of effect sizes. We also included CAge and CAge^2^ as fixed effects to account for nonlinearity in ∆age. We included random intercept terms for birth year and individual ID to account for repeated measures. These models were then repeated using ∆age from the adult morphology clock. All early life modelling was done using the lme4 package (version 1.1-35.5; [31]).

To investigate how ∆age based on morphological traits measured in summer predicted subsequent over-winter survival in adults, we fitted generalized linear mixed-effects models using data from individuals aged 2 years or more. Our response variable was survival to 1 May of the year following phenotypic measurement. An individual was assigned a value of 0 if it died before 1 May and a value of 1 if it survived to 1 May or beyond. We fitted separate models for males and females. We included ∆age and CAge as fixed effects, as well as an interaction between ∆age and CAge to account for the possibility that the effect of ∆age on survival was dependent on CAge. We also included population size in the year of phenotypic measurement and NAO in the winter following phenotypic measurement as covariates since both environmental variables are well-known to have important effects on over-winter survival [27]. Given the known strong association between body weight and over-winter mortality [32], we also fitted weight as a fixed effect in a separate set of models (supplementary material, tables S11–S14) to determine whether any survival signal from the clock primarily reflects variation in body weight. All fixed effects were standardized to a mean of zero and standard deviation of one to facilitate model convergence and comparison of effect sizes. In all models, we included a random intercept term for individual ID and observation year to account for repeated measures. We fitted all models using a Bernoulli distribution (logit link) in the lme4 package (version 1.1-35.5; [31], except for the adult male model which we fitted using a Beta-binomial model (logit link) in the glmmTMB package (version 1.1.9; [33]) to account for overdispersion [34].

## 3. Results

### (a) Phenotypic clock modelling

In both sexes, the morphological clock model predicted age better than models built on either bioassay or behaviour data (figure 2; table 1). In all clocks, there was a general increase in error with age, with a consistent pattern of over-estimation of ages of younger sheep and more pronounced under-estimation of age from around 5 years of age onwards (figure 2). This pattern of over-/under-estimation remained apparent in individuals with repeated records across different ages (supplementary material, figure S3). The phenotypic variables retained for prediction by each model and their estimated effect sizes are reported in the supplementary material, figure S2, also tables S3 and S4.

The morphology clock performed well, predicting age to within half a year (based on MAE) in both females (Pearson: *r* = 0.914, *p* < 0.001; MAE = 0.431; figure 2A) and males (Pearson: *r* = 0.948, *p* < 0.001; MAE = 0.184; table 1; figure 2B). All eight variables were retained in the female model, with 11 variables retained in the male model (supplementary material, figure S2 and table S3). The number of adult teeth had the largest coefficient for both females and males. The bioassay clock had much lower predictive power in both sexes (females: *r* = 0.554, *p* < 0.01; MAE = 1.659; males: *r* = 0.584, *p* < 0.01; MAE = 0.755; table 1). These clocks retained all available variables, with IgE having the largest coefficient in both sexes (supplementary material, figure S2). The behaviour clock performed very poorly in females (*r* = 0.197, *p* < 0.001; MAE = 2.175) and performed comparably to the bioassay clock in males (*r* = 0.604, *p* < 0.001; MAE = 0.929; table 1).

The composite clock including all available data performed comparably to the morphology clock (females: *r* = 0.831, *p* < 0.001; MAE = 0.732; males: *r* = 0.870, *p* < 0.001; MAE = 0.455; table 1; figure 2). The composite clock for females selected 19 of the available 29 variables, with phenotypes coming from all three data sources (supplementary material, figure S2). For males, 20 of the available 33 variables were selected. The composite clock and the morphology clock had similar predictive power, which was surprising given how many more variables could potentially be included in the composite clock. However, the composite clock was based on a much smaller dataset (table 1). To compare age prediction more effectively, we re-ran the morphology clock using the same smaller dataset as the composite clock. The smaller sample morphology clock had very similar predictive power to the composite clock (females: *r* = 0.832, *p* < 0.001, MAE = 0.715; males: *r* = 0.870, *p* < 0.001, MAE = 0.341). This suggests the bioassay and behaviour data added very little additional predictive power. We therefore focussed further analysis on the morphology clock based on all available data to maximize sample size in further analyses. The adult morphological clock (based on data only from individuals 2+ years) also showed appreciable power to predict age (females: *r* = 0.780, *p* < 0.001, MAE = 0.917; males: *r* = 0.817, *p* < 0.001, MAE = 0.525; table 1; supplementary material, figures S4 and S5).

### (b) ∆age, early life conditions, and survival

Repeatability for ∆age from the morphology clock was low but significantly different from zero for both sexes (females: *r* = 0.110, s.e. = 0.019, *p* = 0.000; males: all, *r* = 0.053, s.e. = 0.036, *p* = 0.001). ∆age for adults was predicted by birth weight in both sexes (supplementary material, figures S6 and S7, table S5). Heavier-born individuals had higher ∆age scores (females: estimate = 0.103, s.e. = 0.032, *p* = 0.001; males: estimate = 0.221, s.e. = 0.047, *p* = 0.000). Since our models accounted for the tendency for ∆age to vary with CAge (figure 2), this implies heavy-born individuals tend to have higher ∆age scores independent of CAge. In males only, population size in the year of birth significantly predicted ∆age (estimate = −0.828, s.e. = 0.240, *p* = 0.001; supplementary material, table S5). Males born in years of high resource competition had lower ∆age scores as adults. There was little evidence that twin status, cohort mean faecal egg count, NAO, nor population size in the year before birth influenced ∆age (supplementary material, table S5). When we assessed the effect of early life conditions on ∆age derived from the adult morphology clock, we found comparable effects (supplementary material, table S6).

In adults (ages 2+), ∆age was positively associated with survival in both females (*β* = 0.50 ± 0.15, *z* = 3.41, *p* < 0.001; figure 3A; supplementary material, table S7) and males (*β* = 0.49 ± 0.20, *z* = 2.44, *p* = 0.01; figure 3B; supplementary material, table S8). Survival declined with CAge in females (*β* = −0.84 ± 0.18, *z* = −4.64, *p* < 0.001; supplementary material, table S7) but not in males, once ∆age was accounted for (*β* = −0.29 ± 0.19, *z* = −1.56, *p* = 0.12; supplementary material, table S8). Interactions between age and ∆age were not significant and were removed from final models (females: *β* = −0.002 ± 0.07, *z* = −0.03, *p* = 0.97; males: *β* = 0.07 ± 0.13, *z* = 0.58, *p* = 0.56). In all models, as expected, population size and NAO were negatively associated with survival (supplementary material, tables S7 and S8). However, the effect of ∆age on survival was not independent of body weight, becoming non-significant in models when weight was included (supplementary material, tables S9 and S10). When we assessed the effect of ∆age derived from the adult morphology clock on survival, our results were comparable to those above (supplementary material, figure S8, also tables S11 and S12).

**Figure 3.**
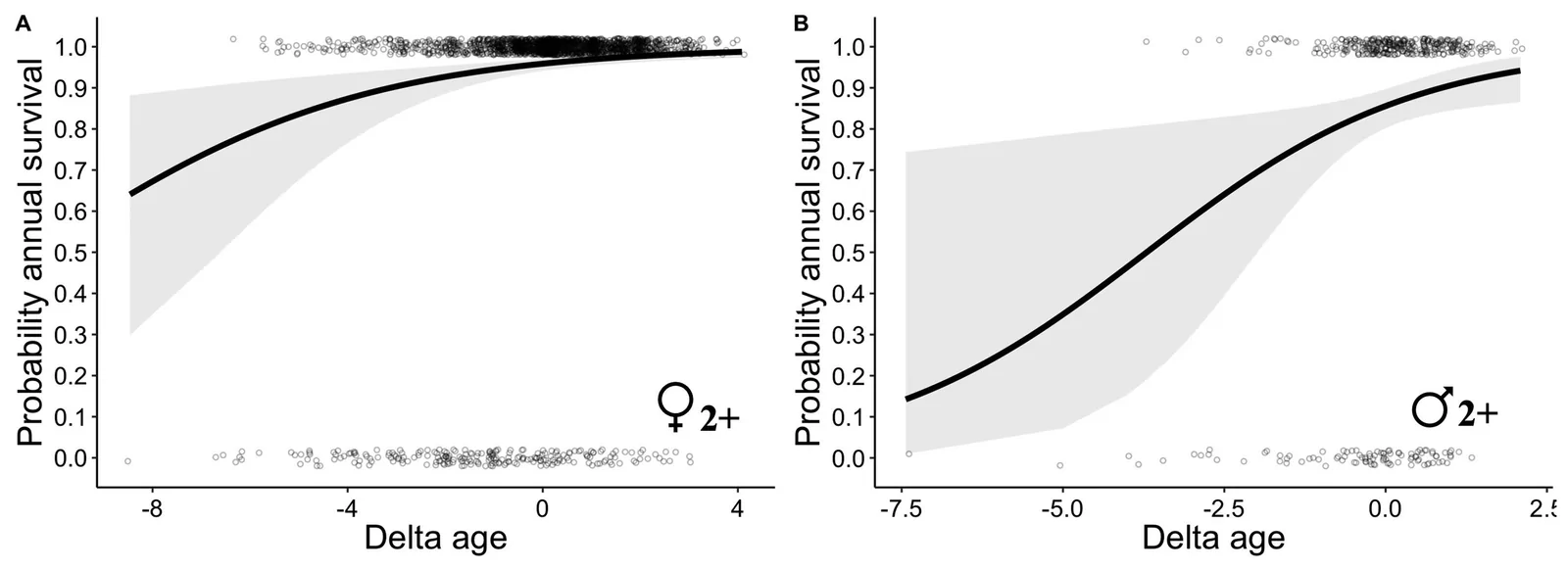
Predicted effects of ∆age on annual survival in (A) adult females (age 2+, *n* = 2113 observations over 730 females), (B) adult males (age 2+; *n* = 423 observations over 228 males). Shaded bars indicate 95% confidence intervals. Points indicate raw data with a small amount of jitter introduced to show overlapping points.

## 4. Discussion

We have shown that CAge can be predicted accurately from a range of morphological phenotypic measures in both sexes in wild Soay sheep. Although it is well-established that epigenetic ageing clock models can accurately predict age across mammal species [15,35] and in some birds [14] and reptiles [36], ours is, to our knowledge, only the second application of ENR models to field-collected *phenotypic* data (see [16]). The previous study used behavioural and non-invasive physiological data from wild baboons to predict age [16]. Their final model had an *r*^2^ of 0.51 (*r* = 0.71) for age prediction, lower than the performance of our Soay sheep female morphological clock (*r* = 0.91). The baboon study did not include morphological data in their analyses and only modelled age in mature adults. On the other hand, we encompassed the full age range, both sexes and a variety of data sources, although restricting data to mature adults only (age 2+ years) produced comparable clock model performance (see table 1). Comparison between our clocks shows that morphological data predicted CAge considerably better than bioassay or behavioural data did in wild Soay sheep. Many of the traits included in the bioassay and behaviour data clocks did change with age in both sexes (see the supplementary material, table S2) [37–39]; however, a clock built using all data predicted age with almost identical accuracy to the morphological clock, suggesting the additional data types contributed minimal predictive power.

There was a clear nonlinear pattern in the age prediction of our clocks, where age was over-estimated in early life and under-estimated in late life (figure 2). This is a pattern seen across many BACs, including the non-invasive baboon clock [16] and wild epigenetic clocks [14,15]. This is often attributed to faster rates of change in early life, particularly in epigenetic clocks [40]. A similar process could explain the pattern in phenotypic clocks if developmental changes captured by the measured traits tend to be faster in early life or are largely complete by mid-adulthood. This is certainly true for traits with the largest effect sizes in our morphological models (e.g. adult tooth number, weight, skeletal size), as changes in these traits after around 4–5 years of age are of much smaller magnitude than those occurring before this point (supplementary material, figure S2). The ability of our clock models to predict age became notably limited at older ages (beyond 7 years in females and 6 years in males, figure 2). This could reflect the limited sample sizes at these ages, or it could reflect the limitations of this type of phenotypic data to accurately predict age in later life as senescence begins. Previous studies have clearly demonstrated that survival and reproductive performance show marked declines with age from around 6 years onwards in Soay sheep [39]. Many of the traits included in our clocks (e.g. weight, parasite egg counts, antibody measures) show opposite directional changes with age in early and later life, improving during development and deteriorating in later adulthood [39]. Given the decreasing sample size for older individuals and the often more pronounced changes in phenotypes during development, it is arguably unsurprising that a phenotypic clock built across all ages would show stronger early life age prediction and carry a stronger signal of development than senescence. However, in adulthood, some phenotypic traits (including weight and IgG) are more strongly predicted by years of life remaining than CAge [22,39]. This implies variation in biological function and senescence that is independent of age, and this variation may further reduce phenotype clocks’ ability to predict age in later life.

Phenotypic deviation from predicted age in our best fitting clock (∆age) was predicted by early life environment and itself predicted subsequent survival, independent of CAge. However, this was in the opposite direction to previously reported ageing clocks. ∆age measures from epigenetic clocks are typically taken as a ‘biological state’ index, with over-estimation reflecting ‘old-for-age’ methylation status and an advanced state of senescence. In human studies, early life adversity is typically found to ‘speed up’ epigenetic clocks and predict increased ∆age [41]. Increased ∆age in adulthood predicts increased mortality risk and poor health outcomes [5]. Weibel *et al.*'s baboon study found that ∆age scores from their phenotype clock produced relationships with early life adversity and survival consistent with this [16]. Notably, they did not include juvenile or morphological data in the clock. Our sheep morphology clock showed the opposite: measures associated with good environmental conditions (being born heavy or in a low competition first year) predicted *greater* ∆age scores. Greater ∆age scores in adults predicted *improved* subsequent winter survival. Together, these patterns suggest that the sheep morphological clock is carrying a strong signal of developmental rate or quality rather than a signal of biological senescence. Although rare, clocks built using early life data appear consistent with our findings. For example, a recent study of wild great tits built an early life epigenetic clock that could accurately predict age over the first few weeks of life [14]. Birds raised in less stressful environments had an elevated epigenetic age [14]. A human epigenetic clock for ages 0–18 years also found increased gestation time elevated epigenetic age [40]. Both patterns are consistent with our sheep morphological clock. However, our clocks were built using data from across all ages, and we analysed ∆age values from adults not juveniles. Further, our ∆age results were unchanged if we used clocks built only from adult data. The findings highlight that ageing clocks are potentially capable of picking up different biological signals depending on the type of data, system, and age range upon which they are built.

There is strong evidence linking early life conditions to developmental rates and quality and, in turn, to adult health or fitness in humans and wild animals [5,26]. These so-called ‘silver spoon’ effects are prevalent in wild animals. Composite phenotypic measures of such developmental variation could be particularly useful in evolutionary ecology, connecting early developmental conditions to integrated adult performance and ultimately fitness [42]. ∆age scores from our morphological clock might provide such an indicator. Adult ∆age scores appear to increase with improved gestational conditions (reflected by heavier birth weight) and predict improved over-winter survival independent of age. However, we note that the effect of ∆age on survival was not independent of body weight, a phenotype included in the clock and known to predict survival in Soay sheep. Interestingly, ∆age and body weight are not strongly correlated in adult sheep (*r* = −0.005, *p* = 0.694). However, the two variables seem to explain overlapping variance in survival probability and may be capturing the same underlying biological process linking early life conditions with adult phenotypic quality and fitness. While these analyses show that ∆age offers little additional explanatory power of survival beyond a single well-established measure of condition in this system, it does show the potential of phenotypic clocks to help measure and understand silver spoon effects in the wild. Not many field studies have the wealth of repeated morphological or other phenotypic data available for our Soay sheep study. Yet it may be possible to combine smaller and sparser field datasets where age is known to build clock models that provide signals of developmental conditions that carry over into adulthood. Inferences from such phenotype clocks could provide important additional predictive power in demographic models and provide insight into between individual differences in condition or state. The next challenge will be to apply comparable phenotype clocks to other populations and species to investigate the generalizability of such models.

We would expect silver spoon effects to persist across lifespan and therefore generate a stable biological signal that remains detectable throughout adulthood. It was striking, therefore, that our ∆age estimates have low repeatability. This also implies low heritability of age acceleration scores, as repeatability typically sets the upper limit for heritability. However, further quantitative genetic analysis would be required to test this. This would suggest that clocks are not capturing a stable, lifelong signature of developmental condition but may reflect something more dynamic or state-dependent. ∆age may capture aspects of both developmental and senescence processes, although the former seems to predominate in our morphological clock. The relative contribution of these processes to ∆age must shift with age, and we expect senescence to be much more stochastic than development owing to the weakening of natural selection with age [2]. This shift and increased physiological and phenotypic stochasticity with age may contribute to the lack of repeatability we observe. This highlights a limitation of producing a clock that spans the entire life course—each phenotype can only be used in ‘one way’ by the model, to either predict development (improvement) or senescence (deterioration). Many phenotypic traits show opposing trends with age during maturation versus late life and probably carry both signals, as discussed above. Despite the importance of identifying and understanding the developmental origins of senescence, a single ageing clock model seems unlikely to produce a ∆age metric that captures and can be related to both processes. An important next step will be to directly test how predictions from phenotypic, epigenetic, and other types of biological ageing clocks are related in wild animals. We are currently assembling an epigenetic clock for Soay sheep and hope to be able to test this in future. Connecting distinct developmental phenotypic clocks (like ours, or those from [14,40]) with epigenetic clocks known to reflect adult functional deterioration within the same system could allow us to empirically link development and senescence.

Population-level phenotypic changes with age in natural populations commonly reflect two opposing processes—within-individual changes with age and selective disappearance. Development and senescence are within-individual processes. Selective disappearance is the progressive loss of lower quality individuals over time, resulting in older age classes that will be disproportionately composed of higher quality survivors [43]. It is a widely observed process that can mask senescence in natural systems without appropriate longitudinal data and statistical correction [9]. This process, which is known to influence age-dependence in many of the phenotypes included in our sheep clock (supplementary material, table S2) might also help explain why our clocks appear to capture a stronger signal of development than senescence, and why many measured traits did not contribute to the clock at all. ENR models built to predict CAge cannot distinguish between within-individual change and among-individual selective filtering. Consequently, for traits in which age-related patterns arise from a combination of individual decline and selective disappearance, these opposing forces may obscure net age trends. In such cases, traits which show important within-individual trends on average may appear weakly associated with CAge, or not associated at all, and therefore contribute little to age prediction. An outstanding challenge is to develop ageing clocks capable of differentiating among- and within-individual processes where longitudinal data are available. Recently, so-called ‘pace of ageing’ clocks have been developed in humans which predict mortality and health outcomes using data on within-individual changes in biomarker and epigenetic data [41,44]. Although they have yet to be applied in non-human systems, they may be more informative for the study of ageing than standard BACs in natural systems where selective disappearance is prevalent.

In conclusion, understanding how developmental processes predict senescence in later life is an important priority for ageing research across disciplines. Epigenetic ageing clocks built using data from adults are the current gold standard for predicting ageing and measuring variation in biological deterioration in humans and wild animals. Our findings suggest that similar clocks built using available phenotypic data collected in the field may not behave in comparable ways to epigenetic clocks, depending on the type of data and age range being used. Our sheep phenotype clock derived almost all its explanatory power of age from morphological traits alone, without inclusion of potentially correlated behavioural or bioassay data. Also it showed much stronger predictive power through early and mid-life than it did in later adulthood. Deviations from clock predictions showed sensitivity to early life environment and prediction of subsequent survival, but in a manner consistent with these scores carrying a signal of developmental rate or quality rather than of senescence. Development and senescence are different processes at opposite ends of life course—while epigenetic clocks clearly reflect the latter process, other types of clocks may detect important variation linked to development and growth. Thus, there could be considerable value in deploying phenotypic and epigenetic clocks in tandem to study questions linked to development and senescence in the wild.

## Supplementary Material



## Data Availability

Data associated with this work and accompanying description can be found on Dryad at [45]. Code associated with the analysis can be found on GitHub at: https://github.com/lrbarn/SoaySheep_PhenotypeClock. Supplementary material is available online [46].

## Theme

One contribution of 21 to a theme issue ‘Developmental circuits in ageing’
