# Supplementary material for: A phenotype-based ageing clock that reflects development rather than senescence in a wild mammal
Source: Philos Trans R Soc Lond B Biol Sci. 2026 Nov 12;381(1963):20250327. doi: 10.1098/rstb.2025.0327 (PMC13755910; doi:10.1098/rstb.2025.0327)
Supplement: Supplementary file 1 [file rstb.2025.0327.zip › rstb.2025.0327/rstb20250327_si_001.docx]

**Supplementary Methods, Tables, and Figures**

**Supplementary Methods**

***Behaviour trait estimation:***

We generated social traits by building sex-specific annual social networks from 1986-2023 using group membership data from the censuses taken in that year. The specifics of network generation are explained in detail elsewhere (see [1]) but briefly, we generated annual association networks using a ‘gambit of the group’ approach [2], where individuals in the same group were taken to be associating. We estimated dyadic associations using the simple ratio index (SRI;[3]), with edge weights ranging from 0 (never seen together) to 1 (never seen apart). Using these annual, sex-specific social networks we calculated ten individual level network measures designed to capture measures of social integration (mean group size, degree, strength, participation coefficient), social bonding (mean strength, social differentiation), and indirect connectedness (eigenvector centrality, clustering, betweenness, closeness; see Table 1). Given that females and males typically associate with members of the same sex outside of the mating season [4], building sex-specific networks was most appropriate. However, we also built annual social networks using data from both sexes and extracted four measures of social integration and bonding with the opposite sex that might capture relevant socio-sexual changes with age (degree, strength, mean strength, social selectivity, see Table 1). To measure home range size, we used kernel density estimation methods in the package adehabitatHR [5] and took the 70th percent isopleth as our estimation of the core home range area [6-8]. Home range size has previously been shown to decline with adult age in the Soay sheep [6]. While detailed analysis of ageing in social network measures has yet to be performed in this system, a growing number of field studies of mammals indicate pronounced changes in social behaviour with age [9] and a previous study of wild baboons found such measures to be important predictors in a phenotypic clock [10].

***Trait changes with Age***

A mixed effect model for each sex and trait was created to test its association with age or age^2^. Individual ID and capture year were included as random effects, following the general formula:

Trait ~ age + age2 + (1|ID) + (1|Year) + ALM.

Age at last measure (ALM) was included to account for selective disappearance. If age^2^ was not significant it was dropped from the model and a linear relationship with age was tested.

***Longitudinal Analysis***

To test how well the best-performing clock – built using morphology data - could accurately predict age across an individual’s lifetime, we capitalised on the 706 females and 205 males who had more than one measure across their lifetime. We measured the correlation between CAge and PAge for individuals that were captured more than once. These data were separated by age at first capture to investigate the performance of the clock across the full lifespan.

The correlation between predicted change and chronological change in both females and males decreased from age at first capture for individuals that were caught more than once (females: r = 0.91 to r = -0.15; males: r = 0.94 to r = 0.25; Figure S3). This suggests that there is a stronger signal of change in early life than in later life, indicating a development rather than senescence clock.

We calculated the chronological and predicted age change between sequential repeated measures from an individual to indicate whether phenotypic change of an individual resulted in an over- or under-estimated change in age. We performed binomial tests to determine whether the morphological clock was more likely than chance (50%) to over- or under-estimate the time elapsed between measures. A binomial test was carried out for all measurement pairs (t and t+1), separated by sex (Females: n = 2076 from 705 individuals, Males: n = 427 from 151 individuals). A “success” in the binomial test was when the predicted change was greater than the chronological change (P_over-estimate_).

Across all repeated measures, the predicted age change was less than the chronological age change significantly more often than the null expectation (females: P_over-esitmate_ = 0.401, p < 2.2e-16; males: P_over-estimate_ = 0.433, p = 0.007). The morphological clock tended to under-estimate change in age between repeated measures of an individual.

**Supplementary Figures**

**Figure S1**: **Change with age for all traits included in clock models.** Each point is an observation. Lines are the linear model or generalised linear model for binomially distributed traits.Females are triangles, males are circles.

**Figure S2: Variable selection and coefficient sizes for each clock and sex** iteration, for coefficient values see Table S3. A) Morphology Clock, B) BioAssay Clock, C) Behaviour Clock, D) Composite Clock. Crosses indicate where a coefficient was not selected by ether the male or female model. All variables were standardised. OS – opposite sex, SS – same sex. Variables are ordered by female coefficient size.

**Figure S3**: **Longitudinal relationship between Phenotypic Age and Chronological Age in individuals with repeated measures.** The number in the panel strip represents the age of the animal at the first measurement. The x axis is the change in years since the first measurement, and the y-axis is the change in phenotypic age. In each panel the blue line is the regression slope, and shading is the standard error, dashed line is y = x. Longitudinal trends show that as animals get older, phenotypic age changes more slowly than chronological age.

**Figure S4: Female non-developmental morphology clock**. Dashed line is x=y, solid line is the linear model of predicted age against chronological age. Pearson’s: r = 0.780, p<2.2e-16, MAE = 0.917

**Figure S5: Male non-developmental morphology clock.** Dashed line is x=y, solid line is the linear model of predicted age against chronological age. Pearson’s: r = 0.817, p<2.2e-16, MAE = 0.525.

**Figure S6: Effect of birth weight on ∆age for adult (2+ years) females,** n = 1549 from 471 individuals. Solid line and shading show the model prediction and 95% confidence interval.

**Figure S7: Effect of birth weight on ∆age for adult (2+ years) males**, n = 294, from 139 individuals. Solid line and shading show the model prediction and 95% confidence interval.

**Figure S8. Survival models for ∆Age from the non-developmental morphology clock.** Results from models fitted with ∆age values calculated from adult-only (2+) phenotype clocks. Predicted effects of ∆age on annual survival in **(A)** adult females (n =2113 observations over 730 females), **(B)** adult males (n = 423 observations over 228 males). Shaded bars indicate 95% confidence intervals. Points indicate raw data with a small amount of jitter introduced to show overlapping points.

**Supplementary Tables**

**Table S1. Table of Soay sheep phenotypes included in each data group, the scale on which that trait was measured, and whether the trait was present in males, females, or both.** All continuous data was z-scored.

| Data group | Variable name | Definition | Scale and units | Male or Female Trait |
| --- | --- | --- | --- | --- |
| Morphological | Unshed Wool? | Coverage of neck, shoulders, back, flanks and rump in unshed wool | 0 = no unshed wool to 25%  1 = more than 25% coverage of unshed wool | M/F |
|  | Scouring? | Whether an individual is showing any signs of current or recent scouring | 0 = no scouring  1 = current or recent scouring | M/F |
|  | Milk present? | Whether a female is currently or has been recently lactating | 0 = not lactating  1 = currently or recently lactating | F |
|  | Teeth Deformed? | Whether an individual has any teeth deformities, such as particularly worn or twisted, or missing teeth | 0 = no deformities  1 = deformities | M/F |
|  | Broken Horns? | Whether one or both horns are broken | 0 = no break  1 = one or both horns broken | M |
|  | Weight | Body mass | Continuous (kg) | M/F |
|  | Foreleg length | Length of the left foreleg | Continuous (mm) | M/F |
|  | Ked count | Count of the number of keds (ectoparasite) on and around belly | Continuous (count) | M/F |
|  | Incisor count | The number of fully erupted pairs of adult incisors present | Continuous (count) | M/F |
|  | Horn Length | The total length of the left horn | Continuous (mm) | M |
|  | Horn Circumference | The circumference of the left horn at the base | Continuous (mm) | M |
|  | Testes Circumference | Scrotal circumference | Continuous (mm) | M |
|  | Testes Length | Scrotal length | Continuous (mm) | M |
| Bioassay | Strongyles | Strongyles parasite eggs in faecal sample | Continuous (number of eggs) | M/F |
|  | Coccidia | Coccidia parasite oocysts in faecal sample | Continuous (number of eggs) | M/F |
|  | IgA | Anti-strongyle parasite antibody levels in blood of IgA isotype | Continuous (corrected optical density score) | M/F |
|  | IgE | Anti-strongyle parasite antibody levels in blood of IgE isotype | Continuous (corrected optical density score) | M/F |
|  | IgG | Anti-strongyle parasite antibody levels in blood of IgG isotype | Continuous (corrected optical density score) | M/F |
|  | RTL | Relative Telomere Length in white blood cells | Continuous (relative telomere length to control gene qPCR score) | M/F |
| Behaviour | Home range size | The spatial area regularly used by an individual, estimated as the 70% isopleth of the utilization distribution. | Continuous (hectares) | M/F |
|  | Mean group size | The average number of same-sex partners an individual associates with per observed grouping event. | Continuous; measure of social integration | M/F |
| Behaviour | Degree | An individual’s number of unique partners. | Continuous; measure of social integration; calculated both for same-sex (SS) interaction and opposite-sex (OS) interaction | M/F |
|  | Strength | The sum of an individual’s dyadic edge weights, which measures how frequently an individual associates with others. | Continuous; measure of social integration; calculated both for same-sex (SS) and opposite-sex (OS) | M/F |
|  | Participation coefficient | A measure of how well distributed an individual’s social associates are among different modules of the network. | Continuous; measure of social integration | M/F |
|  | Mean strength | The average of an individual’s dyadic edge weights, which measures how frequently an individual associates with their partners, on average. | Continuous; measure of social bonding; calculated both for same-sex (SS) and opposite-sex (OS) | M/F |
|  | Social differentiation | The coefficient of variation in an individual’s non-zero dyadic edge weights, which measures how differentiated an individual’s social associations are. | Continuous; measure of social bonding; calculated both for same-sex (SS) and opposite-sex (OS) | M/F |
|  | Eigenvector centrality | Measures how well connected an individual is in the network to individuals who are themselves well connected. | Continuous; measure of indirect connectedness | M/F |
|  | Betweenness | Calculates the number of shortest paths between others in a network that passes through an individual. Individuals with high betweenness have high capacity for linking discrete clusters in the network. | Continuous; measure of indirect connectedness | M/F |
|  | Closeness | Measures the inverse distance to all other individuals in the network. The higher an individual’s closeness score, the easier it is for them to reach others or to be reached. | Continuous; measure of indirect connectedness | M/F |
|  | Clustering | Measures cliquishness or sub-grouping. A high clustering coefficient indicates that an individual’s partners are highly connected to each other. | Continuous; measure of indirect connectedness | M/F |

**Table S2**: Results from models assessing the effect of age on traits included in the phenotypic clock, see supplementary methods. Model of male scouring failed to converge.

| Phenotype |  |  |  | Fixed Effects | | |  | Random Effects | | | Sample Size | |
| --- | --- | --- | --- | --- | --- | --- | --- | --- | --- | --- | --- | --- |
|  | Sex | Error Distribution |  | Age | Age^2^ | Age Last Measure |  | ID | Capture Year | Residual | Observations | Individuals |
| Unshed Wool | Female | Binomial | Estimate | 1.229 | -0.075 | -0.005 | Variance | 73.466 | 1.403 |  | 2725 | 1336 |
|  |  |  | Standard Error | 0.163 | 0.013 | 0.106 | Standard Deviation | 8.571 | 1.184 |  |  |  |
|  |  |  | P Value | 0.000 | 0.000 | 0.959 |  |  |  |  |  |  |
| Unshed Wool | Male | Binomial | Estimate | 0.130 | --- | 0.110 | Variance | --- | 0.690 |  | 1545 | 1080 |
|  |  |  | Standard Error | 0.126 | --- | 0.097 | Standard Deviation | --- | 0.831 |  |  |  |
|  |  |  | P Value | 0.301 | --- | 0.255 |  |  |  |  |  |  |
| Scouring | Female | Binomial | Estimate | -0.354 | 0.065 | -0.311 | Variance | 230.483 | 5.016 |  | 2761 | 1338 |
|  |  |  | Standard Error | 0.237 | 0.023 | 0.144 | Standard Deviation | 15.180 | 2.240 |  |  |  |
|  |  |  | P Value | 0.136 | 0.004 | 0.030 |  |  |  |  |  |  |
| Milk | Female | Binomial | Estimate | 2.610 | -0.202 | -0.029 | Variance | 1.140 | 0.404 |  | 2744 | 1336 |
|  |  |  | Standard Error | 0.129 | 0.011 | 0.031 | Standard Deviation | 1.068 | 0.635 |  |  |  |
|  |  |  | P Value | <2e-16 | <2e-16 | 0.347 |  |  |  |  |  |  |
| Teeth Deformities | Female | Binomial | Estimate | 1.191 | -0.055 | -0.063 | Variance | 2.730 | 0.291 |  | 4913 | 2141 |
|  |  |  | Standard Error | 0.076 | 0.006 | 0.028 | Standard Deviation | 1.652 | 0.540 |  |  |  |
|  |  |  | P Value | <2e-16 | <2e-16 | 0.027 |  |  |  |  |  |  |
| Teeth Deformities | Male | Binomial | Estimate | 2.474 | -0.180 | -0.457 | Variance | 136.910 | 2.950 |  | 2619 | 1817 |
|  |  |  | Standard Error | 0.003 | 0.003 | 0.004 | Standard Deviation | 11.701 | 1.717 |  |  |  |
|  |  |  | P Value | <2e-16 | <2e-16 | <2e-16 |  |  |  |  |  |  |
| Broken Horns | Male | Binomial | Estimate | 0.107 | --- | -0.151 | Variance | 490.300 | 22.140 |  | 2633 | 1831 |
|  |  |  | Standard Error | 0.147 | --- | 0.253 | Standard Deviation | 0.000 | 0.000 |  |  |  |
|  |  |  | P Value | 0.467 | --- | 0.550 |  |  |  |  |  |  |
| Weight | Female | Gaussian | Estimate | 0.535 | -0.037 | 0.058 | Variance | 0.114 | 0.026 | 0.106 | 4982 | 2162 |
|  |  |  | Standard Error | 0.006 | 0.001 | 0.003 | Standard Deviation | 0.338 | 0.162 | 0.326 |  |  |
|  |  |  | P Value | <2e-16 | <2e-16 | <2e-16 |  |  |  |  |  |  |
| Weight | Male | Gaussian | Estimate | 0.860 | -0.072 | 0.047 | Variance | 0.057 | 0.025 | 0.065 | 2662 | 1837 |
|  |  |  | Standard Error | 0.011 | 0.002 | 0.005 | Standard Deviation | 0.240 | 0.159 | 0.255 |  |  |
|  |  |  | P Value | <2e-16 | <2e-16 | <2e-16 |  |  |  |  |  |  |
| Foreleg | Female | Gaussian | Estimate | 0.378 | -0.028 | 0.076 | Variance | 0.316 | 0.145 | 0.178 | 4582 | 2008 |
|  |  |  | Standard Error | 0.008 | 0.001 | 0.005 | Standard Deviation | 0.562 | 0.380 | 0.422 |  |  |
|  |  |  | P Value | <2e-16 | <2e-16 | <2e-16 |  |  |  |  |  |  |
| Foreleg | Male | Gaussian | Estimate | 0.675 | -0.068 | 0.082 | Variance | 0.224 | 0.153 | 0.133 | 2441 | 1705 |
|  |  |  | Standard Error | 0.017 | 0.002 | 0.009 | Standard Deviation | 0.474 | 0.391 | 0.365 |  |  |
|  |  |  | P Value | <2e-16 | <2e-16 | <2e-16 |  |  |  |  |  |  |
| Keds | Female | Gaussian | Estimate | -0.284 | 0.021 | -0.038 | Variance | 1.028 | 0.004 | 0.414 | 4696 | 2040 |
|  |  |  | Standard Error | 0.012 | 0.001 | 0.008 | Standard Deviation | 1.014 | 0.063 | 0.643 |  |  |
|  |  |  | P Value | < 2e-16 | < 2e-16 | 0.000 |  |  |  |  |  |  |
| Keds | Male | Gaussian | Estimate | -1.789 | 0.195 | -0.031 | Variance | --- | 0.302 | 0.550 | 2483 | 1734 |
|  |  |  | Standard Error | 0.098 | 0.014 | 0.034 | Standard Deviation | --- | 7.599 | 2.757 |  |  |
|  |  |  | P Value | <2e-16 | <2e-16 | 0.368 |  |  |  |  |  |  |
| Teeth | Female | Gaussian | Estimate | 0.565 | -0.030 | -0.007 | Variance | 0.002 | 0.002 | 0.084 | 4925 | 2141 |
|  |  |  | Standard Error | 0.004 | 0.000 | 0.001 | Standard Deviation | 0.042 | 0.039 | 0.289 |  |  |
|  |  |  | P Value | < 2e-16 | < 2e-16 | 0.000 |  |  |  |  |  |  |
| Teeth | Maths | Gaussian | Estimate | 0.698 | -0.027 | -0.011 | Variance | --- | 0.005 | 0.115 | 2621 | 1818 |
|  |  |  | Standard Error | 0.012 | 0.002 | 0.004 | Standard Deviation | --- | 0.067 | 0.339 |  |  |
|  |  |  | P Value | <2e-16 | <2e-16 | 0.007 |  |  |  |  |  |  |
| Horn Length | Male | Gaussian | Estimate | 0.856 | -0.070 | 0.010 | Variance | 0.145 | 0.015 | 0.050 | 2580 | 1802 |
|  |  |  | Standard Error | 0.011 | 0.002 | 0.006 | Standard Deviation | 0.381 | 0.122 | 0.224 |  |  |
|  |  |  | P Value | <2e-16 | <2e-16 | 0.108 |  |  |  |  |  |  |
| Horn Circ | Male | Gaussian | Estimate | 0.791 | -0.079 | 0.029 | Variance | 0.317 | 0.068 | 0.111 | 2416 | 1679 |
|  |  |  | Standard Error | 0.017 | 0.002 | 0.010 | Standard Deviation | 0.563 | 0.261 | 0.334 |  |  |
|  |  |  | P Value | < 2e-16 | < 2e-16 | 0.003 |  |  |  |  |  |  |
| Bol Circ | Male | Gaussian | Estimate | 0.755 | -0.074 | 0.073 | Variance | 0.180 | 0.053 | 0.188 | 2197 | 1580 |
|  |  |  | Standard Error | 0.021 | 0.003 | 0.010 | Standard Deviation | 0.424 | 0.230 | 0.434 |  |  |
|  |  |  | P Value | < 2e-16 | < 2e-16 | 0.000 |  |  |  |  |  |  |
| Bol Length | Male | Gaussian | Estimate | 0.758 | -0.077 | 0.070 | Variance | 0.096 | 0.075 | 0.251 | 1584 | 1159 |
|  |  |  | Standard Error | 0.025 | 0.003 | 0.010 | Standard Deviation | 0.310 | 0.273 | 0.501 |  |  |
|  |  |  | P Value | < 2e-16 | < 2e-16 | 0.000 |  |  |  |  |  |  |
| Strongyles | Female | Gaussian | Estimate | -0.228 | 0.017 | -0.079 | Variance | 0.840 | 0.014 | 0.261 | 2660 | 1172 |
|  |  |  | Standard Error | 0.013 | 0.001 | 0.008 | Standard Deviation | 0.917 | 0.117 | 0.511 |  |  |
|  |  |  | P Value | <2e-16 | <2e-16 | <2e-16 |  |  |  |  |  |  |
| Strongyles | Male | Gaussian | Estimate | -0.185 | 0.017 | -0.074 | Variance | 0.746 | 0.062 | 0.318 | 1270 | 908 |
|  |  |  | Standard Error | 0.038 | 0.005 | 0.019 | Standard Deviation | 0.864 | 0.249 | 0.564 |  |  |
|  |  |  | P Value | 0.000 | 0.001 | 0.000 |  |  |  |  |  |  |
| Coccidia | Female | Gaussian | Estimate | -0.150 | 0.011 | -0.033 | Variance | 0.454 | 0.009 | 0.356 | 2660 | 1172 |
|  |  |  | Standard Error | 0.014 | 0.001 | 0.007 | Standard Deviation | 0.674 | 0.095 | 0.596 |  |  |
|  |  |  | P Value | < 2e-16 | 0.000 | 0.000 |  |  |  |  |  |  |
| Coccidia | Male | Gaussian | Estimate | -0.297 | 0.031 | -0.013 | Variance | 0.315 | 0.047 | 0.474 | 1270 | 908 |
|  |  |  | Standard Error | 0.040 | 0.006 | 0.016 | Standard Deviation | 0.561 | 0.218 | 0.689 |  |  |
|  |  |  | P Value | 0.000 | 0.000 | 0.429 |  |  |  |  |  |  |
| IgA | Female | Gaussian | Estimate | 0.304 | -0.021 | 0.031 | Variance | 0.422 | 0.019 | 0.303 | 3828 | 11579 |
|  |  |  | Standard Error | 0.011 | 0.001 | 0.006 | Standard Deviation | 0.650 | 0.139 | 0.550 |  |  |
|  |  |  | P Value | < 2e-16 | < 2e-16 | 0.000 |  |  |  |  |  |  |
| IgA | Male | Gaussian | Estimate | 0.669 | -0.076 | 0.019 | Variance | 0.307 | 0.018 | 0.378 | 1963 | 1328 |
|  |  |  | Standard Error | 0.031 | 0.004 | 0.013 | Standard Deviation | 0.554 | 0.135 | 0.615 |  |  |
|  |  |  | P Value | < 2e-16 | < 2e-16 | 0.144 |  |  |  |  |  |  |
| IgE | Female | Gaussian | Estimate | 0.402 | -0.028 | 0.044 | Variance | 0.266 | 0.014 | 0.253 | 3825 | 1575 |
|  |  |  | Standard Error | 0.010 | 0.001 | 0.005 | Standard Deviation | 0.516 | 0.117 | 0.503 |  |  |
|  |  |  | P Value | <2e-16 | <2e-16 | <2e-16 |  |  |  |  |  |  |
| IgE | Male | Gaussian | Estimate | 0.755 | -0.076 | 0.031 | Variance | 0.161 | 0.006 | 0.295 | 1966 | 1329 |
|  |  |  | Standard Error | 0.025 | 0.004 | 0.010 | Standard Deviation | 0.401 | 0.078 | 0.543 |  |  |
|  |  |  | P Value | < 2e-16 | < 2e-16 | 0.003 |  |  |  |  |  |  |
| IgG | Female | Gaussian | Estimate | 0.402 | -0.028 | 0.044 | Variance | 0.266 | 0.014 | 0.253 | 3825 | 1571 |
|  |  |  | Standard Error | 0.010 | 0.001 | 0.005 | Standard Deviation | 0.516 | 0.117 | 0.503 |  |  |
|  |  |  | P Value | <2e-16 | <2e-16 | <2e-16 |  |  |  |  |  |  |
| IgG | Male | Gaussian | Estimate | 0.755 | -0.076 | 0.031 | Variance | 0.161 | 0.006 | 0.295 | 1966 | 1329 |
|  |  |  | Standard Error | 0.025 | 0.004 | 0.010 | Standard Deviation | 0.401 | 0.078 | 0.543 |  |  |
|  |  |  | P Value | < 2e-16 | < 2e-16 | 0.003 |  |  |  |  |  |  |
| Home Range | Female | Gaussian | Estimate | -0.049 | --- | 0.019 | Variance | 0.513 | 0.482 | 0.338 | 5101 | 1220 |
|  |  |  | Standard Error | 0.004 | --- | 0.008 | Standard Deviation | 0.716 | 0.694 | 0.581 |  |  |
|  |  |  | P Value | < 2e-16 | --- | 0.013 |  |  |  |  |  |  |
| Home Range | Male | Gaussian | Estimate | 0.086 | --- | 0.002 | Variance | 0.289 | 0.351 | 0.491 | 1592 | 653 |
|  |  |  | Standard Error | 0.014 | --- | 0.016 | Standard Deviation | 0.538 | 0.592 | 0.701 |  |  |
|  |  |  | P Value | 0.000 | --- | 0.891 |  |  |  |  |  |  |
| Mean Group Size | Female | Gaussian | Estimate | 0.027 | -0.004 | -0.009 | Variance | 0.2804 | 0.3567 | 0.4303 | 5101 | 1120 |
|  |  |  | Standard Error | 0.012 | 0.001 | 0.007 | Standard Deviation | 0.5295 | 0.5973 | 0.6560 |  |  |
|  |  |  | P Value | 0.032 | 0.000 | 0.183 |  |  |  |  |  |  |
| Mean Group Size | Male | Gaussian | Estimate | 0.609 | -0.055 | 0.002 | Variance | 0.2345 | 0.1836 | 0.4604 | 1592 | 653 |
|  |  |  | Standard Error | 0.038 | 0.005 | 0.015 | Standard Deviation | 0.4843 | 0.4285 | 0.6785 |  |  |
|  |  |  | P Value | < 2e-16 | < 2e-16 | 0.913 |  |  |  |  |  |  |
| Degree SS | Female | Gaussian | Estimate | -0.020 | --- | 0.003 | Variance | 0.406 | 0.360 | 0.389 | 5101 | 1120 |
|  |  |  | Standard Error | 0.004 | --- | 0.007 | Standard Deviation | 0.637 | 0.600 | 0.624 |  |  |
|  |  |  | P Value | 0.000 | --- | 0.649 |  |  |  |  |  |  |
| Degree SS | Male | Gaussian | Estimate | 0.272 | -0.026 | 0.048 | Variance | 0.229 | 0.423 | 0.461 | 1592 | 653 |
|  |  |  | Standard Error | 0.038 | 0.005 | 0.015 | Standard Deviation | 0.479 | 0.650 | 0.679 |  |  |
|  |  |  | P Value | 0.000 | 0.000 | 0.001 |  |  |  |  |  |  |
| Degree OS | Female | Gaussian | Estimate | -0.002 | --- | 0.004 | Variance | 0.082 | 0.459 | 0.503 | 5101 | 1120 |
|  |  |  | Standard Error | 0.004 | --- | 0.005 | Standard Deviation | 0.287 | 0.677 | 0.709 |  |  |
|  |  |  | P Value | 0.663 | --- | 0.472 |  |  |  |  |  |  |
| Degree OS | Male | Gaussian | Estimate | -0.573 | 0.043 | 0.024 | Variance | 0.120 | 0.162 | 0.536 | 1592 | 653 |
|  |  |  | Standard Error | 0.039 | 0.005 | 0.014 | Standard Deviation | 0.346 | 0.403 | 0.732 |  |  |
|  |  |  | P Value | <2e-16 | <2e-16 | 0.080 |  |  |  |  |  |  |
| Strength SS | Female | Gaussian | Estimate | 0.041 | -0.005 | -0.009 | Variance | 0.289 | 0.334 | 0.442 | 5101 | 1120 |
|  |  |  | Standard Error | 0.012 | 0.001 | 0.007 | Standard Deviation | 0.537 | 0.578 | 0.665 |  |  |
|  |  |  | P Value | 0.001 | 0.000 | 0.164 |  |  |  |  |  |  |
| Strength SS | Male | Gaussian | Estimate | 0.619 | -0.057 | 0.007 | Variance | 0.225 | 0.166 | 0.469 | 1592 | 653 |
|  |  |  | Standard Error | 0.038 | 0.005 | 0.015 | Standard Deviation | 0.474 | 0.407 | 0.685 |  |  |
|  |  |  | P Value | <2e-16 | <2e-16 | 0.643 |  |  |  |  |  |  |
| Strength OS | Female | Gaussian | Estimate | -0.003 | --- | -0.002 | Variance | 0.163 | 0.383 | 0.452 | 5101 | 1120 |
|  |  |  | Standard Error | 0.004 | --- | 0.006 | Standard Deviation | 0.404 | 0.619 | 0.672 |  |  |
|  |  |  | P Value | 0.545 | --- | 0.690 |  |  |  |  |  |  |
| Strength OS | Male | Gaussian | Estimate | -0.642 | 0.053 | -0.012 | Variance | 0.260 | 0.172 | 0.327 | 1592 | 653 |
|  |  |  | Standard Error | 0.033 | 0.004 | 0.014 | Standard Deviation | 0.510 | 0.415 | 0.571 |  |  |
|  |  |  | P Value | <2e-16 | <2e-16 | 0.389 |  |  |  |  |  |  |
| Part Coef SS | Female | Gaussian | Estimate | -0.003 | --- | 0.013 | Variance | 0.262 | 0.138 | 0.616 | 5099 | 1120 |
|  |  |  | Standard Error | 0.005 | --- | 0.007 | Standard Deviation | 0.512 | 0.372 | 0.785 |  |  |
|  |  |  | P Value | 0.603 | --- | 0.064 |  |  |  |  |  |  |
| Part Coef SS | Male | Gaussian | Estimate | -0.052 | --- | 0.047 | Variance | 0.095 | 0.398 | 0.723 | 1588 | 652 |
|  |  |  | Standard Error | 0.016 | --- | 0.015 | Standard Deviation | 0.308 | 0.631 | 0.851 |  |  |
|  |  |  | P Value | 0.001 | --- | 0.001 |  |  |  |  |  |  |
| Mean Strength SS | Female | Gaussian | Estimate | 0.026 | -0.002 | -0.010 | Variance | 0.418 | 0.270 | 0.367 | 5101 | 1120 |
|  |  |  | Standard Error | 0.012 | 0.001 | 0.007 | Standard Deviation | 0.646 | 0.520 | 0.606 |  |  |
|  |  |  | P Value | 0.028 | 0.015 | 0.155 |  |  |  |  |  |  |
| Mean Strength SS | Male | Gaussian | Estimate | 0.434 | -0.040 | -0.016 | Variance | 0.213 | 0.476 | 0.540 | 1592 | 653 |
|  |  |  | Standard Error | 0.041 | 0.005 | 0.015 | Standard Deviation | 0.462 | 0.690 | 0.735 |  |  |
|  |  |  | P Value | <2e-16 | 0.000 | 0.288 |  |  |  |  |  |  |
| Mean Strength OS | Female | Gaussian | Estimate | -0.010 | --- | -0.003 | Variance | 0.167 | 0.273 | 0.604 | 5030 | 1116 |
|  |  |  | Standard Error | 0.005 | --- | 0.006 | Standard Deviation | 0.408 | 0.522 | 0.777 |  |  |
|  |  |  | P Value | 0.044 | --- | 0.649 |  |  |  |  |  |  |
| Mean Strength OS | Male | Gaussian | Estimate | -0.488 | 0.042 | -0.036 | Variance | 0.304 | 0.330 | 0.373 | 1578 | 651 |
|  |  |  | Standard Error | 0.036 | 0.004 | 0.015 | Standard Deviation | 0.551 | 0.575 | 0.610 |  |  |
|  |  |  | P Value | <2e-16 | <2e-16 | 0.020 |  |  |  |  |  |  |
| Social Differentiation SS | Female | Gaussian | Estimate | -0.039 | 0.004 | -0.014 | Variance | 0.322 | 0.074 | 0.615 | 5099 | 1120 |
|  |  |  | Standard Error | 0.015 | 0.001 | 0.007 | Standard Deviation | 0.567 | 0.271 | 0.785 |  |  |
|  |  |  | P Value | 0.008 | 0.003 | 0.049 |  |  |  |  |  |  |
| Social Differentiation SS | Male | Gaussian | Estimate | 0.342 | -0.039 | 0.031 | Variance | 0.229 | 0.052 | 0.649 | 1580 | 650 |
|  |  |  | Standard Error | 0.043 | 0.005 | 0.016 | Standard Deviation | 0.479 | 0.229 | 0.805 |  |  |
|  |  |  | P Value | 0.000 | 0.000 | 0.058 |  |  |  |  |  |  |
| Social Differentiation OS | Female | Gaussian | Estimate | -0.002 | --- | -0.006 | Variance | 0.181 | 0.102 | 0.672 | 4790 | 1105 |
|  |  |  | Standard Error | 0.005 | --- | 0.007 | Standard Deviation | 0.425 | 0.319 | 0.819 |  |  |
|  |  |  | P Value | 0.768 | --- | 0.379 |  |  |  |  |  |  |
| Social Differentiation OS | Male | Gaussian | Estimate | -0.654 | 0.052 | -0.040 | Variance | 0.209 | 0.047 | 0.382 | 1560 | 650 |
|  |  |  | Standard Error | 0.035 | 0.004 | 0.014 | Standard Deviation | 0.457 | 0.218 | 0.619 |  |  |
|  |  |  | P Value | < 2e-16 | < 2e-16 | 0.004 |  |  |  |  |  |  |
| Eigen | Female | Gaussian | Estimate | 0.026 | -0.003 | -0.010 | Variance | 0.205 | 0.117 | 0.686 | 5101 | 1120 |
|  |  |  | Standard Error | 0.015 | 0.001 | 0.007 | Standard Deviation | 0.453 | 0.343 | 0.828 |  |  |
|  |  |  | P Value | 0.082 | 0.038 | 0.137 |  |  |  |  |  |  |
| Eigen | Male | Gaussian | Estimate | 0.403 | -0.036 | 0.012 | Variance | 0.076 | 0.141 | 0.735 | 1592 | 653 |
|  |  |  | Standard Error | 0.044 | 0.005 | 0.014 | Standard Deviation | 0.275 | 0.376 | 0.857 |  |  |
|  |  |  | P Value | < 2e-16 | 0.000 | 0.421 |  |  |  |  |  |  |
| Betweenness | Female | Gaussian | Estimate | -0.012 | --- | 0.002 | Variance | 0.068 | 0.046 | 0.846 | 5101 | 1120 |
|  |  |  | Standard Error | 0.006 | --- | 0.006 | Standard Deviation | 0.260 | 0.214 | 0.920 |  |  |
|  |  |  | P Value | 0.027 | --- | 0.778 |  |  |  |  |  |  |
| Betweenness | Male | Gaussian | Estimate | 0.140 | -0.017 | 0.017 | Variance | 0.066 | 0.121 | 0.853 | 1592 | 653 |
|  |  |  | Standard Error | 0.047 | 0.006 | 0.015 | Standard Deviation | 0.256 | 0.348 | 0.924 |  |  |
|  |  |  | P Value | 0.003 | 0.003 | 0.251 |  |  |  |  |  |  |
| Close | Female | Gaussian | Estimate | 0.001 | --- | -0.004 | Variance | 0.024 | 2.268 | 0.045 | 5099 | 1120 |
|  |  |  | Standard Error | 0.001 | --- | 0.002 | Standard Deviation | 0.156 | 1.506 | 1.506 |  |  |
|  |  |  | P Value | 0.638 | --- | 0.063 |  |  |  |  |  |  |
| Close | Male | Gaussian | Estimate | 0.083 | -0.008 | -0.001 | Variance | 0.003 | 3.262 | 0.059 | 1588 | 652 |
|  |  |  | Standard Error | 0.012 | 0.001 | 0.004 | Standard Deviation | 0.059 | 1.806 | 0.243 |  |  |
|  |  |  | P Value | 0.000 | 0.000 | 0.895 |  |  |  |  |  |  |
| Clustering | Female | Gaussian | Estimate | 0.014 | --- | -0.011 | Variance | 0.279 | 0.130 | 0.618 | 5099 | 1120 |
|  |  |  | Standard Error | 0.005 | --- | 0.007 | Standard Deviation | 0.528 | 0.360 | 0.786 |  |  |
|  |  |  | P Value | 0.005 | --- | 0.130 |  |  |  |  |  |  |
| Clustering | Male | Gaussian | Estimate | 0.269 | -0.020 | -0.029 | Variance | 0.104 | 0.231 | 0.749 | 1580 | 650 |
|  |  |  | Standard Error | 0.046 | 0.005 | 0.015 | Standard Deviation | 0.322 | 0.481 | 0.865 |  |  |
|  |  |  | P Value | 0.000 | 0.000 | 0.059 |  |  |  |  |  |  |

**Table** **S3**: **Coefficients of terms included in morphology, bioassay, and behaviour clocks (all ages).** Terms shaded grey were not fitted as they are absent in one sex. --- represents terms that were absent from the final clock.

|  |  |  | **Composite** |  |
| --- | --- | --- | --- | --- |
| **Variable** | Female | Male | Female | Male |
| ***Morphology*** |  |  |  |  |
| (Intercept) | 3.248 | 1.094 | 3.351 | 1.519 |
| Unshed Wool | 0.346 | 0.080 | 0.193 | --- |
| Scouring | 0.383 | 0.005 | --- | --- |
| Teeth Deformity | 0.883 | 0.259 | 0.595 | --- |
| Weight | 0.255 | 0.325 | 0.361 | 0.050 |
| Foreleg | 0.096 | --- | --- | 0.023 |
| Teeth | 2.543 | 1.045 | 2.322 | 1.029 |
| Keds | -0.072 | -0.053 | --- | --- |
| Milk | -0.590 |  | -0.527 |  |
| Horn Length |  | 0.540 |  | 0.850 |
| Horn Circumference |  | -0.229 |  | -0.601 |
| Bol Circumference |  | -0.009 |  | --- |
| Bol Length |  | 0.001 |  | 0.088 |
| Broken Horns |  | 0.148 |  | --- |
| ***BioAssay*** |  |  |  |  |
| *(intercept)* | 3.297 | 1.285 |  |  |
| Strongyles | -0.704 | -0.184 | 0.020 | -0.055 |
| Coccidia | -0.198 | -0.229 | --- | --- |
| IgA | 0.667 | 0.143 | 0.122 | -0.069 |
| IgE | 1.058 | 0.689 | 0.070 | -0.029 |
| IgG | -0.126 | 0.306 | --- | -0.060 |
| RTL | -0.094 | -0.167 | 0.018 | -0.031 |
| ***Behaviour*** |  |  |  |  |
| *(intercept)* | 4.485 | 2.766 |  |  |
| Home Range Size | -0.343 | 0.048 | -0.068 | 0.264 |
| same sex deg | -0.084 | 0.066 | --- | 0.045 |
| same sex strength | 2.293 | 0.468 | --- | -0.038 |
| same sex eigen | -0.085 | --- | -0.000 | -0.026 |
| same sex cluster | 0.494 | -0.022 | 0.110 | --- |
| same sex between | -0.049 | -0.115 | -0.098 | -0.050 |
| same sex close | -0.002 | 0.095 | --- | --- |
| same sex part coef | 0.402 | 0.017 | 0.102 | --- |
| same sex mean strength | -0.467 | -0.065 | 0.022 | -0.240 |
| same sex mean group size | -2.157 | -0.205 | -0.221 | --- |
| same sex social differentiation | -0.162 | 0.021 | 0.032 | -0.070 |
| opposite sex degree | 0.041 | -0.705 | --- | --- |
| opposite sex strength | -0.150 | 0.393 | --- | 0.064 |
| opposite sex mean strength | -0.001 | -0.581 | -0.005 | --- |
| opposite sex social differentiation | -0.061 | -0.558 | 0.039 | -0.162 |

**Table S4**: **Coefficients retained in the adult morphology clock (2 years and older).** Traits shaded grey are absent in that sex. --- represents terms that were absent from the final clock.

| **Variable** | Female | Male |
| --- | --- | --- |
| ***Morphology Clock*** |  |  |
| *(Intercept)* | 3.077 | 1.150 |
| Unshed Wool | 0.329 | --- |
| Scouring | 0.688 | --- |
| Teeth Deformity | 0.891 | 0.258 |
| Weight | 0.314 | 0.310 |
| Foreleg | 0.105 | --- |
| Teeth | 2.700 | 1.121 |
| Keds | -0.110 | --- |
| Milk | -0.644 |  |
| Horn Length |  | 0.626 |
| Horn Circumference |  | -0.500 |
| Bol Circumference |  | --- |
| Bol Length |  | --- |
| Broken Horns |  | 0.130 |

**Table S5: Fixed and random effects for early life experience models for adults (2+ years).** Significant terms are in bold, all values rounded to 3 decimal places. The male model failed to converge with all random effects fitted, so Birth Year was removed.

|  | Female | | Male |  |
| --- | --- | --- | --- | --- |
|  | Adult (n=1548, from 471 individuals) | | Adult (n=293, from 139 individuals) | |
| *Fixed effects* | *Estimate*  *(Std. Err)* | *P-value* | *Estimate*  *(Std. Err)* | *P-value* |
| (Intercept) | **-0.403**  **(0.134)** | **0.003** | **-0.927**  **(0.175)** | **0.000** |
| NAO | 0.030  (0.014) | 0.056 | -0.016  (0.014) | 0.252 |
| Birth Weight | **0.120**  **(0.038)** | **0.002** | **0.265**  **(0.056)** | **0.000** |
| Singleton (Yes) | -0.180  (0.098) | 0.066 | -0.096  (0.130) | 0.461 |
| Population Size  (birth year) | 0.018  (0.221) | 0.934 | **-0.825**  **(0.240)** | **0.001** |
| Population Size (year before birth) | 0.384  (0.198) | 0.073 | 0.085  (0.234) | 0.717 |
| Cohort mean FEC | -0.055  (0.051) | 0.307 | -0.040  (0.050) | 0.425 |
| CAge | **0.711**  **(0.036)** | **< 2e-16** | **0.843**  **(0.065)** | **< 2e-16** |
| CAge^2^ | **-0.093**  **(0.003)** | **< 2e-16** | **-0.133**  **(0.007)** | **< 2e-16** |
| *Random Effects* | *Variance* | *Std. dev.* | *Variance* | *Std. dev.* |
| ID | 0.074 | 0.273 | 0.076 | 0.275 |
| Birth Year | 0.019 | 0.137 | --- | --- |
| Residual | 0.743 | 0.862 | 0.195 | 0.442 |

**Table S6**: **Fixed and random effects for early life experience models based on ∆age from the adult morphology clock**. Significant terms are in bold, all values rounded to 3 decimal places.

|  | Female | | Male | |
| --- | --- | --- | --- | --- |
|  | (n=1548, from 471 individuals) | | (n=293, from 139 individuals) | |
| *Fixed effects* | *Estimate*  *(Std. Err)* | *P-value* | *Estimate*  *(Std. Err)* | *P-value* |
| (Intercept) | **0.774**  **(0.144)** | **<0.001** | **-131**  **(0.189)** | **<0.001** |
| NAO | 0.033  (0.015) | 0.051 | -0.010  (0.015) | 0.532 |
| Birth Weight | **0.135**  **(0.041)** | **0.001** | **0.290**  **(0.062)** | **<0.001** |
| Singleton (Yes) | -0.186  (0.105) | 0.078 | -0.185  (0.144) | 0.203 |
| Population Size  (birth year) | 0.017  (0.238) | 0.945 | **0.687**  **(0.265)** | **0.011** |
| Population Size (year before birth) | 0.404  (0.213) | 0.080 | 0.230  (0.260) | 0.378 |
| Cohort mean FEC | -0.063  (0.055) | 0.276 | -0.061  (0.055) | 0.274 |
| CAge | **0.810**  **(0.038)** | **<0.001** | **0.929**  **(0.069)** | **<0.001** |
| CAge^2^ | **-0.098**  **(0.003)** | **<0.001** | **-0.138**  **(0.007)** | **<0.001** |
| *Random Effects* | *Variance* | *Std. dev.* | *Variance* | *Std. dev.* |
| ID | 0.090 | 0.299 | 0.105 | 0.324 |
| Birth Year | 0.022 | 0.148 |  | Singular |
| Residual | 0.838 | 0.915 | 0.216 | 0.464 |

**Table S7.** **Fixed and random effects from adult female survival model** (ages 2+). All regression coefficients are standardized, and significant effects are indicated in bold. (n = 2112 observations over 730 females).

| Effect | Group | Term | Estimate | Std error | Test-statistic | P value |
| --- | --- | --- | --- | --- | --- | --- |
| **Fixed Effects** |  | Intercept | 3.16 | 0.27 | 11.90 | <0.001 |
|  |  | **Delta age** | **0.50** | **0.15** | **3.41** | **<0.001** |
|  |  | **CAge** | **-0.84** | **0.18** | **-4.64** | **<0.001** |
|  |  | **NAO** | **-0.65** | **0.18** | **-3.54** | **<0.001** |
|  |  | **Population size** | **-0.85** | **0.19** | **-4.43** | **<0.001** |
| **Random Effects** | Individual.ID | sd(intercept) | 0.95 |  |  |  |
|  | Year | sd(intercept) | 0.65 |  |  |  |

**Table S8.** **Fixed and random effects from adult male survival model** (ages 2+). All regression coefficients are standardized, and significant effects are indicated in bold. (n = 422 observations over 228 males).

| Effect | Group | Term | Estimate | Std error | Test-statistic | P value |
| --- | --- | --- | --- | --- | --- | --- |
| **Fixed Effects** |  | Intercept | 1.79 | 0.20 | 9.17 | <0.001 |
|  |  | **Delta age** | **0.49** | **0.20** | **2.44** | **0.01** |
|  |  | CAge | -0.29 | 0.19 | -1.56 | 0.12 |
|  |  | **NAO** | **-0.96** | **0.20** | **-4.77** | **<0.001** |
|  |  | **Population size** | **-1.25** | **0.22** | **-5.64** | **<0.001** |
| **Random Effects** | Individual.ID | sd(intercept) | 0.0001 |  |  |  |
|  | Year | sd(intercept) | 0.3 |  |  |  |

**Table S9.** **Fixed and random effects from the adult female survival model (ages 2+) including weight**. Regression coefficients are standardized, and significant effects are indicated in bold (n = 2112 observations over 730 females).

| Effect | Group | Term | Estimate | Std error | Test-statistic | P value |
| --- | --- | --- | --- | --- | --- | --- |
| **Fixed Effects** |  | Intercept | 2.06 | 0.27 | 7.64 | <0.001 |
|  |  | Delta age | 0.17 | 0.15 | 1.14 | 0.25 |
|  |  | **CAge** | **-1.61** | **0.29** | **-5.53** | **<0.001** |
|  |  | **Weight** | **2.43** | **0.40** | **6.07** | **<0.001** |
|  |  | **NAO** | **-0.65** | **0.22** | **-2.98** | **0.003** |
|  |  | **Population size** | **-0.80** | **0.22** | **-3.60** | **<0.001** |
| **Random Effects** | Individual.ID | sd(intercept) | 1.08 |  |  |  |
|  | Year | sd(intercept) | 0.79 |  |  |  |

**Table S10.** **Fixed and random effects from the adult male survival model (ages 2+) including weight**. Regression coefficients are standardized, and significant effects are indicated in bold (n = 422 observations over 228 males).

| Effect | Group | Term | Estimate | Std error | Test-statistic | P value |
| --- | --- | --- | --- | --- | --- | --- |
| **Fixed Effects** |  | Intercept | 1.21 | 0.24 | 5.09 | <0.001 |
|  |  | Delta age | -0.07 | 0.24 | -0.28 | 0.78 |
|  |  | **CAge** | **-1.38** | **0.32** | **-4.33** | **<0.001** |
|  |  | **Weight** | **0.22** | **0.05** | **4.38** | **<0.001** |
|  |  | **NAO** | **-0.94** | **0.22** | **-4.20** | **<0.001** |
|  |  | **Population size** | **-1.21** | **0.24** | **-4.98** | **<0.001** |
| **Random Effects** | Individual.ID | sd(intercept) | 0.0001 |  |  |  |
|  | Year | sd(intercept) | 0.41 |  |  |  |

**Table S11.** **Fixed and random effects from female survival model fitted with delta age values calculated from the adult morphology clock.** Regression coefficients are standardized, and significant effects are indicated in bold (n = 2112 observations over 730 females).

| Effect | Group | Term | Estimate | Std error | Test-statistic | P value |
| --- | --- | --- | --- | --- | --- | --- |
| **Fixed Effects** |  | Intercept | 3.16 | 0.27 | 11.87 | <0.001 |
|  |  | **Delta age** | **0.49** | **0.14** | **3.59** | **<0.001** |
|  |  | **Age** | **-0.87** | **0.18** | **-4.92** | **<0.001** |
|  |  | **NAO** | **-0.65** | **0.18** | **-3.55** | **<0.001** |
|  |  | **Population size** | **-0.85** | **0.19** | **-4.43** | **<0.001** |
| **Random Effects** | Individual.ID | sd(intercept) | 0.96 |  |  |  |
|  | Year | sd(intercept) | 0.65 |  |  |  |

**Table S12.** **Fixed and random effects from male survival model fitted with delta age values calculated from the adult morphology clock.** Regression coefficients are standardized, and significant effects are indicated in bold (n = 423 observations over 228 males).

| Effect | Group | Term | Estimate | Std error | Test-statistic | P value |
| --- | --- | --- | --- | --- | --- | --- |
| **Fixed Effects** |  | Intercept | 1.80 | 0.20 | 9.21 | <0.001 |
|  |  | **Delta age** | **0.49** | **0.19** | **2.56** | **0.01** |
|  |  | Age | -0.32 | 0.18 | -1.82 | 0.07 |
|  |  | **NAO** | **-0.97** | **0.20** | **-4.82** | **<0.001** |
|  |  | **Population size** | **-1.25** | **0.22** | **-5.67** | **<0.001** |
| **Random Effects** | Individual.ID | sd(intercept) | 0.0001 |  |  |  |
|  | Year | sd(intercept) | 0.29 |  |  |  |

**Supplementary References**

[1] Siracusa, E. R., Bal, X., De Moor, D., Albery, G. F., Beattie, R., Ravindran, S., Wiersma, E., Wilson, A. R., Pemberton, J. M., Nussey, D. H., et al. 2025 Environment dependent benefits of sociality in Soay sheep. *bioRxiv*. (DOI:10.1101/2025.05.04.652099).

[2] Franks, D. W., Ruxton, G. D. & James, R. 2009 Sampling animal association networks with the gambit of the group. *Behavioral Ecology and Sociobiology* **64**, 493–503. (DOI:10.1007/s00265-009-0865-8).

[3] Whitehead, H. 2008 Precision and power in the analysis of social structure using associations. *Animal Behaviour* **75**, 1093–1099. (DOI:10.1016/j.anbehav.2007.08.022).

[4] Clutton-Brock, T. & Pemberton, J. 2004 *Soay sheep: dynamics and selection in an island population.* Cambridge, UK, Cambridge University Press. .

[5] Calenge, C. 2006 The package “adehabitat” for the R software: A tool for the analysis of space and habitat use by animals. *Ecological Modelling* **197**, 516–519. (DOI:10.1016/j.ecolmodel.2006.03.017).

[6] Froy, H., Borger, L., Regan, C. E., Morris, A., Morris, S., Pilkington, J. G., Crawley, M. J., Clutton-Brock, T. H., Pemberton, J. M. & Nussey, D. H. 2018 Declining home range area predicts reduced late-life survival in two wild ungulate populations. *Ecol Lett* **21**, 1001–1009. (DOI:10.1111/ele.12965).

[7] Wiersma, E., Pakeman, R. J., Bal, X., Pilkington, J. G., Pemberton, J. M., Nussey, D. H. & Sweeny, A. R. 2023 Age-specific impacts of vegetation functional traits on gastrointestinal nematode parasite burdens in a large herbivore. *J Anim Ecol* **92**, 1869–1880. (DOI:10.1111/1365-2656.13978).

[8] Regan, C. E., Pilkington, J. G., Pemberton, J. M. & Crawley, M. J. 2016 Sex differences in relationships between habitat use and reproductive performance in Soay sheep (Ovis aries). *Ecol Lett* **19**, 171–179. (DOI:10.1111/ele.12550).

[9] Siracusa, E. R., Higham, J. P., Snyder-Mackler, N. & Brent, L. J. N. 2022 Social ageing: exploring the drivers of late-life changes in social behaviour in mammals. *Biol Lett* **18**, 20210643. (DOI:10.1098/rsbl.2021.0643).

[10] Weibel, C. J., Dasari, M. R., Jansen, D. A., Gesquiere, L. R., Mututua, R. S., Warutere, J. K., Siodi, L. I., Alberts, S. C., Tung, J. & Archie, E. A. 2024 Using non-invasive behavioral and physiological data to measure biological age in wild baboons. *Geroscience* **46**, 4059–4074. (DOI:10.1007/s11357-024-01157-5).
